# Differential shaping of equine gut microbiota structure and function by breed and feeding regimen

**DOI:** 10.3389/fmicb.2026.1852554

**Published:** 2026-07-16

**Authors:** Yanan Lin, Gere Qiri, Ming Du, Manglai Dugarjaviin, Jialong Cao, Xinlan Fang, Siqin Yun, Xu Li, Wendian Gong, Wenqi Ding, Tugeqin Bou, Shaofeng Su, Jiameng Chen, Na Xing, Dongyi Bai, Yiping Zhao

**Affiliations:** 1 Key Laboratory of Equus Germplasm Innovation (Co-Construction by Ministry and Province), Ministry of Agriculture and Rural Affairs, Inner Mongolia Key Laboratory of Equine Science Research and Technology Innovation, Equus Research Center, College of Animal Science, Inner Mongolia Agricultural University, Hohhot, China; 2 Xing An Polytechnic University, Ulanhot, China; 3 Inner Mongolia Academy of Agricultural & Animal Husbandry Sciences, Hohhot, China

**Keywords:** bacterial diversity, breed, feces, feeding regimen, gut microbiota

## Abstract

**Background:**

The gut microbiota plays an essential role in host energy metabolism and immune function. Horses are non-ruminant herbivores that rely heavily on hindgut microbial fermentation to meet their energy requirements. However, the relative contributions of host genetic background (breed) and environmental factors (feeding regimen and geographical location) to shaping the equine gut microbiota remain poorly understood.

**Methods:**

In this study, 16S rRNA gene sequencing and functional prediction analysis were performed on 139 equine fecal samples to systematically investigate the differential effects of breed and feeding regimen on the gut microbiota. Samples were collected from 30 Thoroughbreds (TH), 31 stabled hybrid horses (HH1), 30 grazing hybrid horses (HH2) (with HH1 and HH2 sired by Thoroughbreds out of Mongolian mares), 32 Mongolian horses (MH), and 16 Warmblood horses (WBH1 and WBH2). Alpha and beta diversity analyses, taxonomic profiling, and PERMANOVA were used to assess microbial composition and the contributions of different factors.

**Results:**

Alpha diversity analysis revealed that the richness and diversity of the TH, HH1, HH2, and MH groups were significantly higher than those of the Warmblood horses (*p* < 0.001), with Mongolian horses exhibiting the highest diversity and the hybrids showing intermediate levels between their parental breeds. Regarding taxonomic composition, the TH, HH1, HH2, and MH groups shared a microbial structure dominated by Firmicutes and Bacteroidota, yet each possessed distinct characteristics: Thoroughbreds were enriched with Treponema; Mongolian horses harbored the highest abundances of Rikenellaceae_RC9_gut_group and NK4A214_group; and the grazing hybrid horses developed a fiber-degrading bacterial community centered on *Ruminococcus* and *Fibrobacter*, demonstrating breed-specific microbial features. In contrast, the Warmblood horses exhibited a gut microbiota with distinct features characterized by significantly reduced microbial diversity and core fiber-degrading genera, concomitant with an enrichment of environmental-associated bacteria from the phylum Proteobacteria (e.g., *Acinetobacter*, *Stenotrophomonas*) and other genera (e.g., *Comamonas*, *Brevundimonas*). PERMANOVA analysis further quantified the contributions of different factors: breed explained 44.8% of the total variation (R² = 0.448, p < 0.001), followed by feeding regimen (10.3%, p < 0.001) and geographical location (2.7%, *p* < 0.01), confirming breed as the predominant factor.

**Conclusion:**

This study provides evidence that breed establishes the foundational framework of the gut microbiota, while feeding regimen performs fine-tuning functions. We also systematically characterized the unique microbial composition of Warmblood horses, offering a scientific basis for breed-specific health management, precision nutritional interventions, and future disease risk monitoring in horses. Although all horses appeared clinically healthy, the distinct microbial composition observed in Warmblood horses warrants further investigation to determine its biological significance.

## 1 Introduction

The gut microbiota plays an essential role in host energy metabolism and immune function (Sun et al., 2024). Accumulating evidence indicates that the microbial community serves the host by extracting energy, stimulating immunity, excluding pathogens, and detoxifying harmful compounds (Jyoti and Dey, 2025). Consequently, it plays a pivotal role in the pathogenesis of various diseases, including obesity (Depommier et al., 2019), diabetes (Frost et al., 2021), non-alcoholic fatty liver disease (Loomba et al., 2019), liver cirrhosis (Trebicka et al., 2021), pancreatic cancer (Riquelme et al., 2019), inflammatory bowel disease (Lloyd-Price et al., 2019), and colorectal cancer (Saus et al., 2019).

Horses are non-ruminant herbivores, and their hindgut (cecum and colon) serves as a fermentation chamber for a complex and dynamic microbial population. The equine diet is rich in fiber, which comprises molecules indigestible by host enzymes. Bacteria residing in the hindgut, particularly those with fibrolytic capabilities, slowly ferment this fiber, enabling the horse to thrive on a high-fiber diet (Shepherd et al., 2012). The microorganisms inhabiting the mammalian intestine possess a far greater arsenal of degradative enzymes and metabolic capabilities than their host. They degrade complex, non-digestible dietary carbohydrates and host-derived glycans in the gut, generating energy that can be utilized by the host. Ruminant herbivores derive up to 80% of their daily caloric intake from microbial fermentation, with an average feed retention time of 57 h (Udén et al., 1982). In horses, volatile fatty acids, the end-products of fiber fermentation, provide more than 50% of their daily energy requirements (Argenzio et al., 1974). Certain dominant bacterial phyla, particularly Bacteroidetes, are known to possess a vast array of genes encoding carbohydrate-active enzymes and can readily switch between different energy sources available in the gut (Flint et al., 2012).

Despite the evident importance of the gut microbiota, a comprehensive and systematic understanding of the equine intestinal microbial community remains lacking. While extensive research has focused on defining a “healthy” gut microbiota and its association with host physiological functions (Hou et al., 2022), our comprehension of what constitutes “normal” versus “abnormal” microbial states is still very limited. Under healthy conditions, the gut microbiota exhibits stability, resilience, and a symbiotic relationship with the host. A healthy microbial community is often characterized by high taxonomic diversity, high microbial gene richness, and a stable core microbiota (Fan and Pedersen, 2021). Establishing the intestinal microbial composition of healthy horses is therefore crucial for assessing the impact of disease.

Due to practical difficulties and ethical constraints associated with sampling the hindgut directly, most research on gut microbiota composition is conducted using fecal samples as a proxy. Several studies have reported that the fecal bacterial community does not significantly differ from that of the colon (Mach et al., 2017), or even the cecum (Mura et al., 2019). The definition of a core microbiota in horses is still under investigation. Some studies have identified the predominant phyla in healthy horses as Firmicutes, Bacteroidetes, Spirochaetes, and Kiritimatiellaeota, with key genera including uncharacterized Lachnospiraceae, Treponema, and others (Chaucheyras-Durand et al., 2022). In summary, research on the gut microbiome is a currently burgeoning field that not only helps elucidate the intricate relationship between the gut microbiome and health but also provides novel insights and strategies for the prevention and treatment of various diseases.

The composition of the commensal gut microbiota in a host is malleable (Jia et al., 2008) and influenced by a multitude of factors, including geographic location, season, breed, age, dietary composition, and variations in management and husbandry practices (Garber et al., 2020). However, the relative contributions of host genetic background (breed) and environmental factors (feeding regimen and geographical location) to shaping the equine gut microbiota remain poorly understood, particularly in the context of the confounding effects that often arise from field sampling designs. Comparing the intestinal microbial composition of horses under different management systems or of different breeds can provide profound insights into the structure and function of the equine gut microbiota, offering valuable references for equine health management and disease prevention.

The present study systematically evaluated the effects of breed, feeding regimen, and geographical location on the gut microbiota of healthy horses. We performed 16S rRNA gene sequencing on fecal samples from 139 horses, encompassing three breeds (Thoroughbred, Mongolian, and Warmblood) and two hybrid groups (Thoroughbred × Mongolian crossbreds), sourced from four distinct equine facilities in Inner Mongolia and Beijing, China. The objectives of this study were to: (1) characterize the gut microbial composition of healthy horses across different breeds and management systems; (2) disentangle the independent contributions of breed, feeding regimen, and geographical location to microbial community structure using multivariate statistical approaches; and (3) identify breed- and management-associated microbial signatures that may inform equine health management. These data contribute to a more detailed characterization of the gut microbiota in healthy horses and provide a valuable microbial resource for advancing equine health management practices.

## 2 Materials and methods

### 2.1 Animals and sample collection

Fresh fecal samples were collected from a total of 139 healthy male horses (aged 2–8 years) representing six groups. The sample cohort comprised: 30 Thoroughbreds (TH, Hohhot, stabled), 31 stabled hybrid horses (HH1, Hohhot, stabled, Thoroughbred × Mongolian crossbred), 30 grazing hybrid horses (HH2, Xilingol League, grazing, Thoroughbred × Mongolian crossbred), 32 Mongolian horses (MH, Xilingol League, grazing), and 16 Warmblood horses (WBH1: 5 from Beijing; WBH2: 11 from Xing’an League, both stabled). All horses were male to avoid gender-related confounding effects on gut microbiota composition. Feeding regimen: Stabled horses (TH, HH1, and WBH) were housed in individual stalls with free access to hay and water. Grazing horses (HH2 and MH) were maintained on natural pastures in Xilingol League with free access to fresh forage and water, with no concentrate supplementation.

All horses had been maintained at four different equine facilities in Inner Mongolia and Beijing, China, for a minimum of 12 months and had not experienced any recent changes in diet or housing conditions prior to sampling. Sampling was conducted between June and September 2020 to control for seasonal variation in gut microbiota. Immediately following natural defecation, fecal samples were collected into sterile tubes, transported to the laboratory in liquid nitrogen, and stored at −80 °C until DNA extraction.

Health status definition: Health status was assessed by licensed veterinarians based on the following criteria: (1) normal body temperature (36–38 °C); (2) absence of clinical signs of respiratory or digestive diseases; (3) no history of antibiotic or anthelmintic use within the past three months; and (4) normal appetite, water intake, and defecation behavior.

Body Condition Score (BCS) assessment: The Henneke BCS system (1–9 scale) was used to evaluate the body condition of all horses by assessing fat coverage across six body regions (neck, withers, shoulder, ribs, back, and tailhead) through visual inspection and palpation (Henneke et al., 1983). In this study, all horses had BCS values ranging from 5 to 7 (ideal to moderately fleshy), indicating good nutritional status.

Detailed information for each horse, including age, BCS, and sampling location, is provided in Supplementary Table S1.

### 2.2 DNA extractions

DNA from different samples was extracted using the E.Z.N.A. ®Stool DNA Kit (D4015, Omega, Inc., USA) according to manufacturer ‘s instructions. The reagent which was designed to uncover DNA from trace amounts of sample has been shown to be effective for the preparation of DNA of most bacteria. Nuclear-free water was used for blank. The total DNA was eluted in 50 μL of Elution buffer and stored at −80 °C until measurement in the PCR by LC-Bio Technology Co., Ltd., Hang Zhou, Zhejiang Province, China.

### 2.3 PCR amplification and 16S rDNA sequencing

The 5′ ends of the primers were tagged with specific barcods per sample and sequencing universal primers. Primer sequence: 341F (5′-CCTACGGGNGGCWGCAG-3′), 805R (5′-GACTACHVGGGTATCTAATCC-3′) (Logue et al., 2016). PCR amplification was performed in a total volume of 25 μL reaction mixture containing 25 ng of template DNA, 12.5 μL PCR Premix, 2.5 μL of each primer, and PCR-grade water to adjust the volume. The PCR conditions to amplify the prokaryotic 16S fragments consisted of an initial denaturation at 98 °C for 30 s; 32 cycles of denaturation at 98 °C for 10 s, annealing at 54 °C for 30 s, and extension at 72 °C for 45 s; and then final extension at 72 °C for 10 min. The PCR products were confirmed with 2% agarose gel electrophoresis. Throughout the DNA extraction and PCR amplification process, negative controls using sterile water instead of template DNA were included in each batch to exclude the possibility of false-positive PCR results and potential contamination. No detectable 16S rRNA amplification products were observed in any negative control. The PCR products were purified by AMPure XT beads (Beckman Coulter Genomics, Danvers, MA, USA) and quantified by Qubit (Invitrogen, USA). The amplicon pools were prepared for sequencing and the size and quantity of the amplicon library were assessed on Agilent 2100 Bioanalyzer (Agilent, USA) and with the Library Quantification Kit for Illumina (Kapa Biosciences, Woburn, MA, USA), respectively. The libraries were sequenced on NovaSeq PE250 platform.

### 2.4 Data analysis

Samples were sequenced on an Illumina NovaSeq platform according to the manufacturer’s recommendations, provided by LC-Bio. Paired-end reads was assigned to samples based on their unique barcode and truncated by cutting off the barcode and primer sequence. Paired-end reads were merged using FLASH. (v1.2.8) Quality filtering on the raw reads were performed under specific filtering conditions to obtain the high-quality clean tags according to the fqtrim (v0.94). Chimeric sequences were filtered using Vsearch software (v2.3.4). After dereplication using DADA2, we obtained feature table and feature sequence. Alpha diversity and beta diversity were calculated by normalized to the same sequences randomly. Then according to SILVA (release 132) classifier, feature abundance was normalized using relative abundance of each sample. Alpha diversity is applied in analyzing complexity of species diversity for a sample through 5 indices, including Chao1, observed species, goods coverage, Shannon, Simpson, and all this indices in our samples were calculated with QIIME2 (Bolyen et al., 2019). Beta diversity were calculated by QIIME2, the graphs were drew by R package. Blast was used for sequence alignment, and the feature sequences were annotated with SILVA database for each representative sequence. Other diagrams were implemented using the R package (v3.5.2).

Based on the obtained species abundance statistics, differential analysis was performed between comparison groups. Different statistical methods were selected according to the sample conditions: the Mann–Whitney *U* test (also known as the Wilcoxon rank-sum test) was used for two-group comparisons with biological replicates; the Kruskal–Wallis test was used for multi-group comparisons with biological replicates. The screening threshold was set at *p* < 0.05. LDA effect size (LEfSe, LDA > 3.0, *p* value <0.05) was performed using nsegata-lefse. Other diagrams were implemented using the R package (v3.4.4). Functional potential was predicted using PICRUSt2 (v2.5.2).

### 2.5 PERMANOVA analysis

In this study, permutational multivariate analysis of variance (PERMANOVA) was used to assess the effects of different factors on the beta diversity of equine gut microbiota. Based on the Bray–Curtis distance matrix, we constructed the following three models to disentangle the independent contributions of breed, management, and geographical location:

Model 1 (Full model):


$$y_{\text{ijkl}}=\mu +\text{Bree}d_{i}+\text{Managemen}t_{j}+\text{Locatio}n_{k}+e_{\text{ijkl}}$$

where $y_{\text{ijkl}}$ is the Bray–Curtis distance matrix between samples; μ is the population mean; $\text{Bree}d_{i}$ is the breed effect (*i* = TH, HH1, HH2, MH, WBH1, WBH2); $\text{Managemen}t_{j}$ is the management effect (*j* = stable-fed, grazing -fed); $\text{Locatio}n_{k}$ is the geographical location effect (*k* = Hohhot, Xilingol League, Beijing, Xing’an League); and $e_{\text{ijkl}}$ is the random error term. All PERMANOVA analyses were performed with 999 permutations to estimate significance levels, and the proportion of variation explained by each factor was quantified using *R*^2^ values.

## 3 Results

### 3.1 Sequencing results

A total of 139 fresh fecal samples were collected from healthy horses (aged 2–8 years) across four distinct geographical locations. Following 16S rRNA gene sequencing of all samples, and after rigorous quality control and sequence optimization, a total of 95,235 amplicon sequence variants (ASVs) were identified from 6,264,986 raw reads. The average sequencing depth per sample was 45,071.84 ± 10,692.81 reads. Detailed data are provided in Supplementary Table S2.

### 3.2 Alpha diversity analysis

The Good’s coverage estimator for all samples ranged from 98.3 to 99.5%, indicating that the sequencing depth was sufficient to capture the bacterial communities present in the fecal samples (Table 1). The results revealed that microbial richness and diversity in the TH, HH1, HH2, and MH groups were significantly higher than those in the Warmblood horses (WBH1 and WBH2 groups) (*p* < 0.001). Although not statistically significant, a consistent trend was observed in alpha diversity among the TH, HH1, HH2, and MH groups. MH groups exhibited the highest values for both Observed Species and Shannon indices, whereas TH groups showed the lowest Shannon index (*p* < 0.001). The hybrid groups (HH1 and HH2) displayed intermediate values.

**Table 1:** Alpha diversity index of different horses.

Breed	Goods_coverage	Observed_species	Shannon	Simpson	Chao1	Pielou_e	Ace
TH	0.983 ± 0.008^b^	1242.6 ± 397.6^a^	9.07 ± 0.56^b^	0.995 ± 0.004^a^	1402.3 ± 451.8^a^	0.885 ± 0.020^b^	1403.3 ± 453.6^a^
HH1	0.984 ± 0.009^b^	1241.5 ± 441.2^a^	9.26 ± 0.67^a^	0.997 ± 0.003^a^	1392.4 ± 497.8^a^	0.901 ± 0.0024^a^	1385.9 ± 499.7^a^
HH2	0.985 ± 0.009^b^	1165.8 ± 403.4^a^	9.22 ± 0.54^a^	0.996 ± 0.0023^a^	1316.7 ± 458.5^a^	0.905 ± 0.0019^a^	1313.8 ± 461.8^a^
MH	0.981 ± 0.010^b^	1313.3 ± 371.3^a^	9.31 ± 0.58^a^	0.997 ± 0.003^a^	1492.3 ± 428.9^a^	0.910 ± 0.0021^a^	1483.5 ± 430.9^a^
WBH1	0.995 ± 0.002^a^	369.2 ± 131.8^b^	5.47 ± 0.34^c^	0.916 ± 0.036^b^	414.3 ± 148.4^b^	0.632 ± 0.0056^c^	418.2 ± 148.2^b^
WBH2	0.994 ± 0.006^a^	407.9 ± 237.7^b^	5.05 ± 1.46^c^	0.877 ± 0.097^b^	462.8 ± 265.6^b^	0.596 ± 0.103^c^	467.6 ± 271.3^b^
*p*-value	< 0.001	< 0.001	< 0.001	< 0.001	< 0.001	< 0.001	< 0.001

Values are presented as mean ± standard deviation. Different superscript letters (a, b, c) within the same column indicate statistically significant differences among groups (*p* < 0.05).

Furthermore, no significant differences in alpha diversity metrics were observed between groups of the same breed, specifically between HH1 and HH2 (hybrids under different management) and between WBH1 and WBH2 (Warmbloods from different locations) (*p* > 0.05). Similarly, comparisons within the same geographical location and management condition revealed no significant differences in microbial community richness and diversity, neither between TH and HH1 (both stabled in Hohhot) nor between MH and HH2 (both grazing in Xilingol) (*p* > 0.05).

### 3.3 Beta diversity analysis

**Figure 1:**
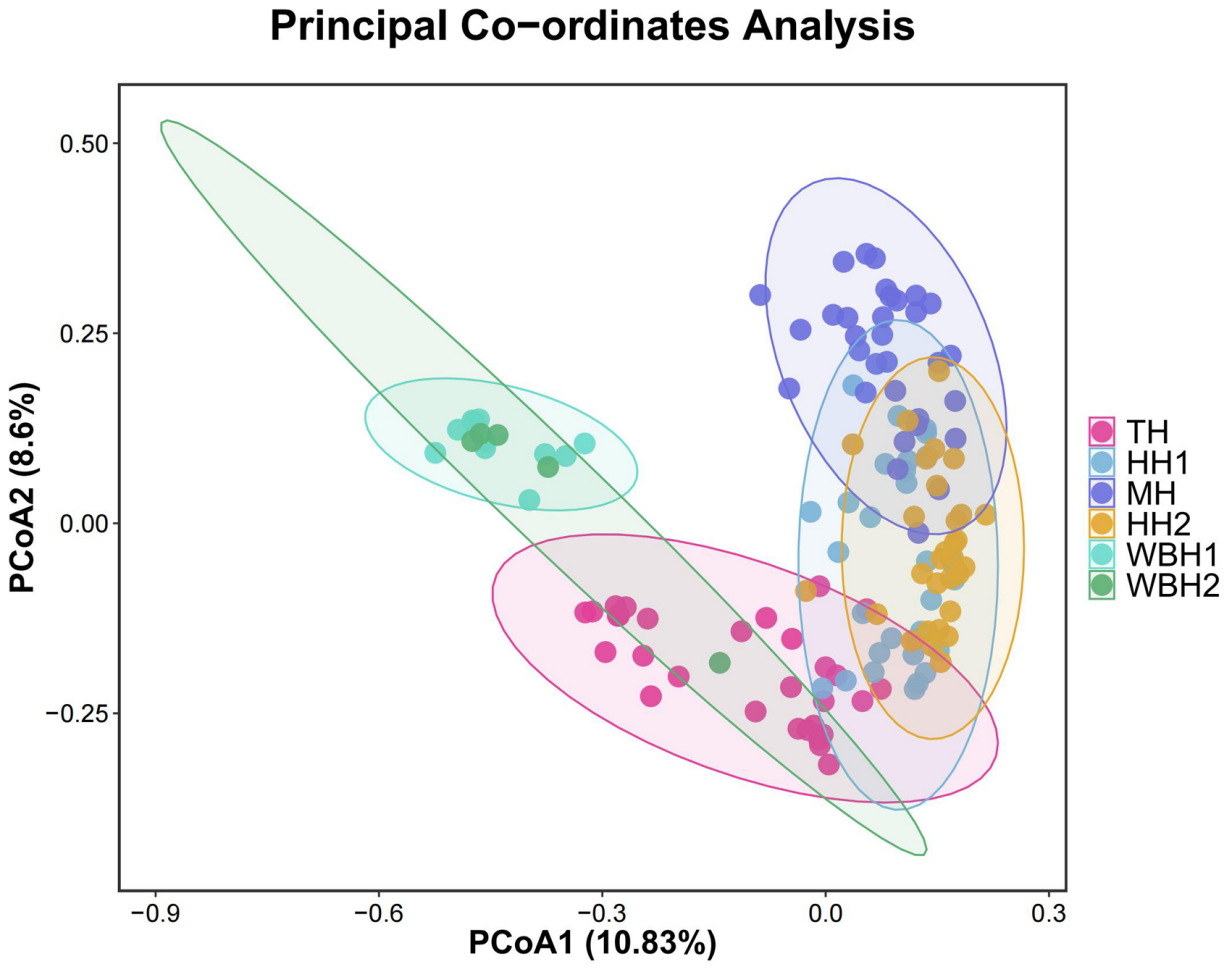
Principal coordinates analysis (PCoA) on Bray–Curtis distance illustrates proximity matrices present in feces of horses.

Principal coordinate analysis (PCoA) based on Bray–Curtis distances revealed distinct clustering patterns among the horse groups (Figure 1). The gut microbiota of Warmblood horses (WBH1 and WBH2) were clearly separated from those of the other four groups (TH, HH1, HH2, and MH) along the first principal coordinate axis (PCoA1), which explained 10.83% of the total variation in microbial community structure (Figure 1). Along the second principal coordinate axis (PCoA2), accounting for 8.6% of the community variation, a clear separation was observed among the TH, HH1, HH2, and MH groups (Figure 1). These results demonstrate that horses with distinct genetic backgrounds—Thoroughbreds, hybrids, Mongolian horses, and Warmbloods—harbor significantly different gut microbial community structures. This finding is consistent with the alpha diversity results.

Permutational multivariate analysis of variance (PERMANOVA) based on Bray–Curtis distances was subsequently employed to quantitatively assess the effects of different factors on microbial community structure. The analysis revealed that all examined factors—breed, feeding regimen, and geographic location—exerted highly significant effects (*p* < 0.01). Among these, breed was the predominant factor, explaining 44.8% of the total microbial community variation (*R*^2^ = 0.448, *p* < 0.001). Feeding regimen accounted for 10.3% of the variation, while geographic location explained 2.7% (Table 2). To ensure that the PERMANOVA results were not biased by differences in within-group dispersion, a permutational analysis of multivariate dispersions (PERMDISP) was performed. The PERMDISP test showed no significant differences in multivariate dispersion among the six groups (*F* = 0.559, *p* = 0.732 > 0.05, Supplementary Figure 1), confirming that the observed compositional differences reflect genuine shifts in microbial community centroids rather than heterogeneity in within-group variability.

**Table 2:** PERMANOVA analysis of equine gut microbiota based on Bray–Curtis distances across five horse groups.

Factors	df	SS	MS	*F*.Model	*R* ^2^	*p*-value
Breed	4	3.8745	0.9686	28.342	**0.448**	**0.001**
Feeding regimen	1	0.8923	0.8923	26.114	**0.103**	**0.001**
Geographic location	2	0.2341	0.1171	3.425	0.027	**0.002**
Residuals	131	4.4768	0.0342	–	0.422	–
Total	138	8.6477	–	–	1.000	–

PERMANOVA, permutational multivariate analysis of variance; df, degrees of freedom; SS, sum of squares; MS, mean squares; *F*.Model, *F*-statistic calculated by the PERMANOVA model; *R*^2^, effect size representing the proportion of total variation explained by each factor, with larger values indicating greater contribution. *p*-values were calculated based on 999 permutations. Bold values indicate significant effects (*p* < 0.05).

To further disentangle the influence of feeding regimen from genetic background, a direct comparison was performed between the HH1 and HH2 groups, which share the same hybrid genetic background but differ in management (stabled vs. grazing). This analysis demonstrated that feeding regimen explained 13.4% of the variation between these two groups, with a highly significant difference (*p* < 0.001), providing direct evidence that feeding practices significantly shape the gut microbial structure under identical genetic backgrounds (Table 3).

**Table 3:** PERMANOVA analysis of groups with the same breed but different feeding regimens (HH1 vs. HH2).

Factors	df	SS	MS	*F*.Model	*R* ^2^	*p*-value
Feeding regimen	1	0.2347	0.2347	8.342	**0.134**	**0.001**
Residuals	59	1.5234	0.0258	–	0.866	–
Total	60	1.7581	–	–	1.000	–

Conversely, a comparison between Warmblood horse groups from different geographic locations (WBH1 vs. WBH2), which share the same breed and similar management (stabled), revealed no significant difference (*R*^2^ = 0.081, *p* = 0.267), indicating that geographic location alone, in the absence of breed and management variation, does not substantially alter the microbial community structure (Table 4).

**Table 4:** PERMANOVA analysis of groups with the same breed but different geographic locations (WBH1 vs. WBH2).

Factors	df	SS	MS	*F*.Model	*R* ^2^	*p*-value
Geographic location	1	0.0452	0.0452	1.234	0.081	0.267
Residuals	14	0.5128	0.0366	–	0.919	–
Total	15	0.5580	–	–	1.000	–

### 3.4 Characteristics of fecal bacterial communities

**Figure 2:**
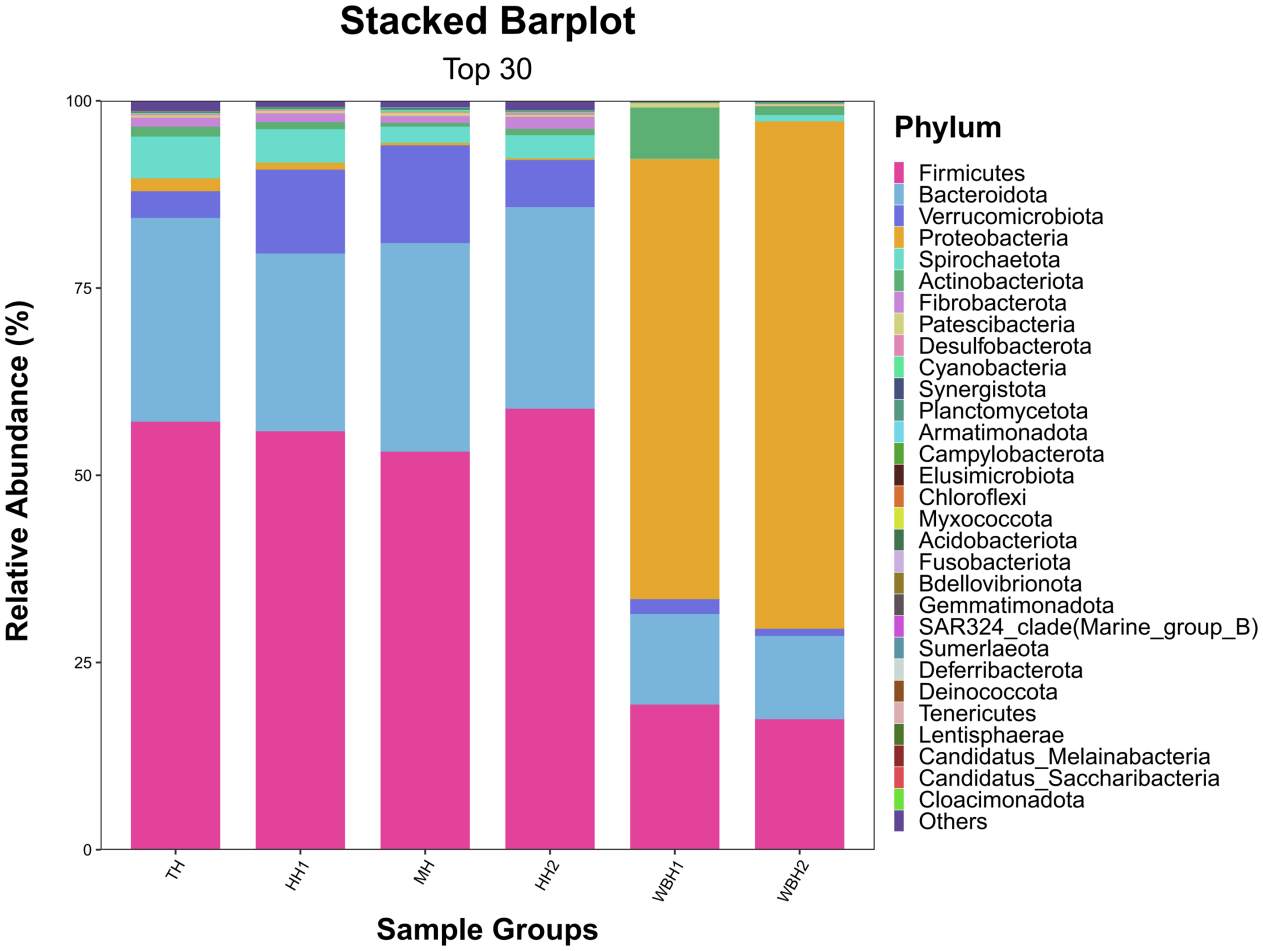
Relative abundance of bacterial groups from the TH, HH1, HH2, MH, and WBH groups at the phylum level.

The relative abundance profiles of the dominant bacterial communities at the phylum levels are illustrated using stacked bar charts in Figure 2. In the TH, HH1, HH2, and MH groups, the predominant bacterial phyla were Firmicutes (53.28–59.01%), Bacteroidota (23.75–27.85%), Verrucomicrobiota (3.55–13.04%), Spirochaetota (2.18–5.59%), and Fibrobacterota (0.91–1.65%). In contrast, the Warmblood horses (WBH1 and WBH2) exhibited a markedly distinct gut microbial composition compared to the other four groups. Their dominant phyla were Proteobacteria (58.76 and 67.75%), Firmicutes (19.50 and 17.55%), Bacteroidota (12.11 and 11.13%), and Actinobacteriota (6.81 and 1.13%), a profile that differed significantly from that of the remaining four groups (*p* < 0.05). Notably, several phyla showed significant differences in relative abundance among the groups (*p* < 0.05). Specifically, Thoroughbreds (TH) harbored the highest relative abundance of Spirochaetota, while Mongolian horses (MH) exhibited the highest relative abundance of Verrucomicrobiota. The hybrid groups (HH1 and HH2) displayed an overall gut microbial structure intermediate between their parental breeds (TH and MH). Within the hybrids, the HH2 group (grazing) showed the highest relative abundances of Firmicutes and Fibrobacterota, suggesting a potentially enhanced capacity for dietary fiber degradation.

**Figure 3:**
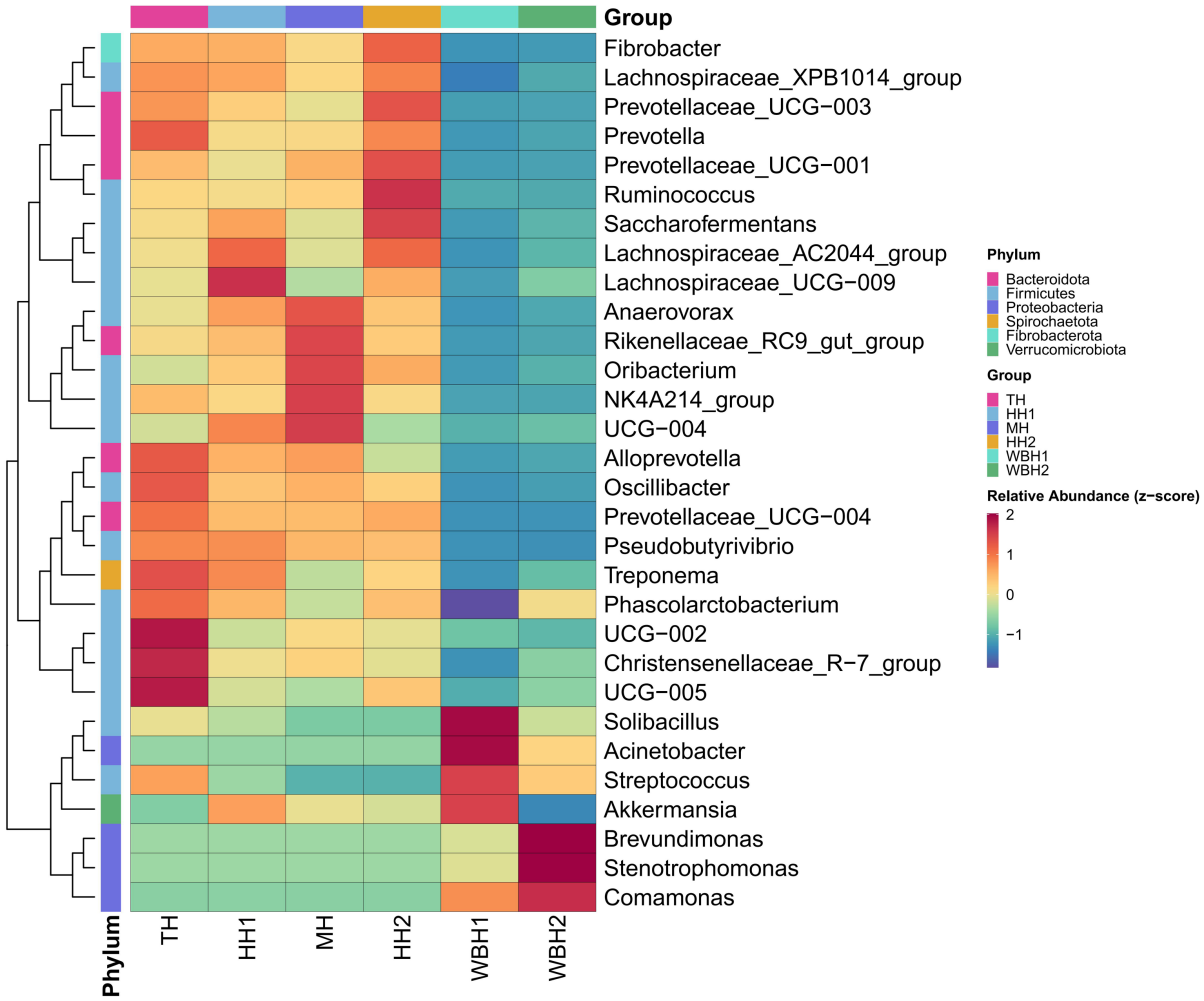
Relative abundance of bacterial groups from the TH, HH1, HH2, MH, and WBH groups at the genus level.

At the genus level (Figure 3), the TH, HH1, HH2, and MH groups shared several dominant genera (relative abundance >2%), including *Rikenellaceae_RC9_gut_group* (4.86–9.68%), *NK4A214_group* (3.95–7.92%), *Treponema* (2.09–5.47%), *Lachnospiraceae_XPB1014_group* (2.97–4.20%), *Lachnospiraceae_AC2044_group* (2.05–4.19%), and *Ruminococcus* (2.29–5.36%). In contrast, the Warmblood horses (WBH1 and WBH2) exhibited a markedly distinct gut microbial composition compared to the other four groups. In the WBH1 group, the dominant genera were *Acinetobacter* (38.74%), *Comamonas* (5.64%), *Solibacillus* (2.78%), *Streptococcus* (2.97%), and *Stenotrophomonas* (2.73%). Notably, the relative abundances of *Rikenellaceae_RC9_gut_group* (0.13%), *Ruminococcus* (0.07%), and *Treponema* (0.06%) in WBH1 were significantly lower than those in the other groups (*p* < 0.01). In the WBH2 group, the dominant genera included *Stenotrophomonas* (17.60%), *Brevundimonas* (16.25%), *Acinetobacter* (11.80%), and *Comamonas* (9.11%). Compared to WBH1, WBH2 exhibited relatively higher abundances of certain genera, such as *Lachnospiraceae_UCG-009* (0.94%) and *Christensenellaceae_R-7_group* (1.36%).

In the TH group, the abundances of *Treponema* (5.47%), *UCG-002* (3.19%), *UCG-005* (2.49%), and *Streptococcus* (1.99%) were significantly higher than those in the other groups (*p* < 0.05). In the HH1 group, the dominant genera were *Lachnospiraceae_UCG-009* (4.87%), *Lachnospiraceae_AC2044_group* (4.19%), *Rikenellaceae_RC9_gut_group* (6.00%), and *Akkermansia* (0.77%). Among these, *Lachnospiraceae_UCG-009* was significantly more abundant in HH1 compared to the other groups (*p* < 0.05). In the MH group, the dominant genera were *Rikenellaceae_RC9_gut_group* (9.68%), *NK4A214_group* (7.92%), *Oribacterium* (0.84%), and *UCG-004* (0.91%). The abundances of *Rikenellaceae_RC9_gut_group* and *NK4A214_group* were significantly higher in MH than in the other groups (*p* < 0.05). In the HH2 group, the abundances of *Ruminococcus* (5.36%), *Saccharofermentans* (2.02%), *Lachnospiraceae_XPB1014_group* (4.20%), *Fibrobacter* (1.60%), and *Prevotellaceae_UCG-001* (1.81%) were significantly higher than those in the other groups (*p* < 0.05).

**Figure 4:**
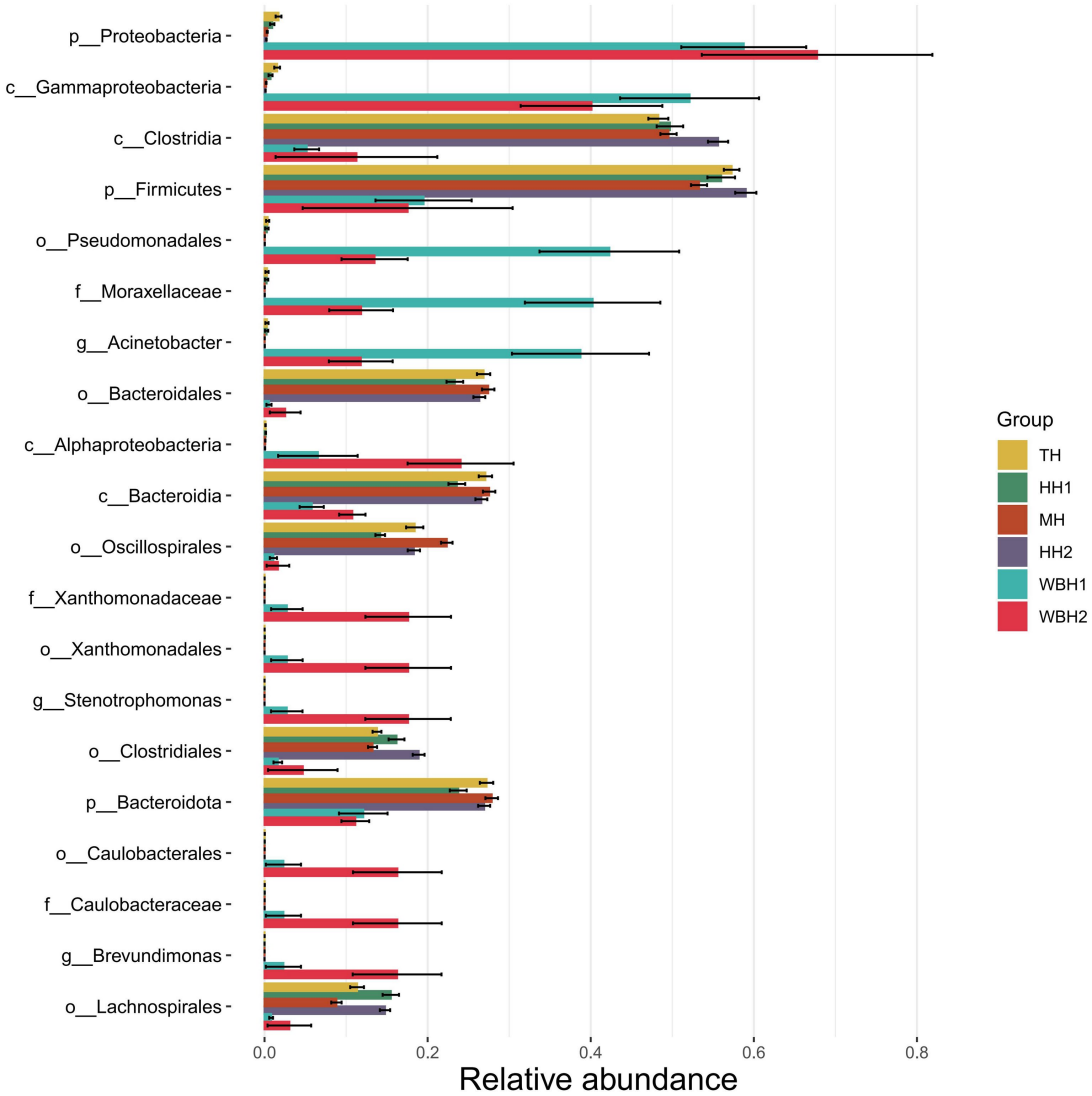
Linear discriminant analysis effect size (LEfSe) of differentially abundant microbial taxa among the six horse groups.

LEfSe (Linear discriminant analysis Effect Size) was employed to identify differentially abundant microbial biomarkers among the six horse groups (Figure 4). The analysis revealed distinct enrichment patterns across the groups. In Thoroughbreds (TH), the following taxa were significantly enriched: *Treponema*, Spirochaetaceae, Spirochaetota, *Phascolarctobacterium*, Acidaminococcaceae, Negativicutes, *UCG-002*, and *UCG-005*. In stabled hybrid horses (HH1), significant enrichment was observed for *Lachnospiraceae_UCG-009*, *Lachnospiraceae_XPB1014_group*, Lachnospiraceae, and Lachnospirales. In grazing hybrid horses (HH2), the following taxa were significantly more abundant compared to the other groups: *Ruminococcus*, Ruminococcaceae, Firmicutes, Clostridia, Clostridiales, *p-251-o5_unclassified*, Prevotellaceae, and *Lachnospiraceae_AC2044_group*. In Mongolian horses (MH), significantly enriched taxa included Verrucomicrobiota, Kiritimatiellaeota, *WCHB1-41_unclassified*, *Rikenellaceae_RC9_gut_group*, Rikenellaceae, *NK4A214_group*, Oscillospiraceae, and *UCG-010*. In WBH1 (Warmblood horses from Beijing), the following taxa were significantly enriched: *Acinetobacter*, Moraxellaceae, Pseudomonadales, Flavobacteriia, *Empedobacter*, Planococcaceae, and *Solibacillus*. In WBH2 (Warmblood horses from the Xing’anmeng), significantly enriched taxa comprised *Brevundimonas*, Caulobacteraceae, Caulobacterales, *Stenotrophomonas*, Xanthomonadaceae, *Comamonas*, and *Sphingobacterium*.

### 3.5 Prediction and comparison of gene function of fecal microbiota

**Figure 5:**
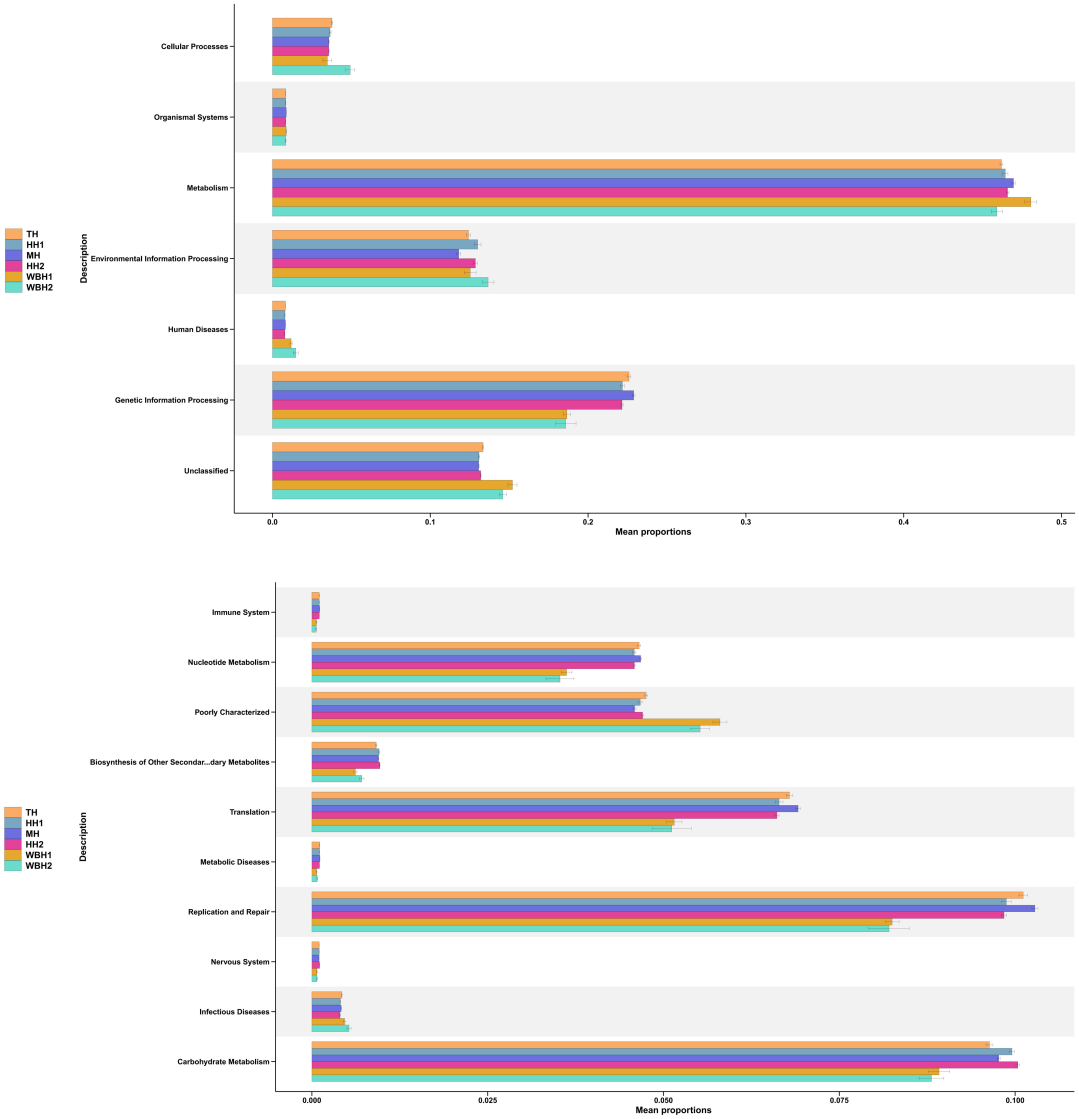
Functional predictions of the equine fecal microbiota using PICRUSt2 at KEGG Level 1 and Level 2.

Functional predictions of the equine fecal microbiota were performed using PICRUSt2, yielding gene function annotations at KEGG Level 2 (Figure 5). The results revealed that the major functional modules (relative abundance >0.05) across the six horse groups included Membrane Transport, Amino Acid Metabolism, Carbohydrate Metabolism, Replication and Repair, and Translation. Despite these shared functional profiles, significant differences in specific metabolic pathways were observed among the groups. Notably, the HH2 group (grazing hybrids) exhibited the highest capacity for Carbohydrate Metabolism, while the MH group (Mongolian horses) demonstrated the most pronounced capabilities in Amino Acid Metabolism and Energy Metabolism. In contrast, the WBH1 and WBH2 groups (Warmblood horses) showed significantly lower levels of Carbohydrate Metabolism, Replication and Repair, Translation, and Nucleotide Metabolism compared to the other four groups (*p* < 0.05). Conversely, these Warmblood groups exhibited significantly higher levels of Xenobiotics Biodegradation and Metabolism, Lipid Metabolism, Signal Transduction, and Cellular Processes and Signaling relative to the other four groups (*p* < 0.05).

## 4 Discussion

The gut microbiota, widely recognized as the host’s “second genome” (Qin et al., 2010), plays a pivotal role in nutrient metabolism, immune regulation, and disease defense (Clemente et al., 2012; Bergamaschi et al., 2020). Its composition is influenced by multiple factors, including breed (Bergamaschi et al., 2020), host genetics (Lu et al., 2018), diet, and environment (López-García et al., 2021). The interplay between host phylogeny and feeding habits has been demonstrated to jointly shape mammalian gut microbiota composition, with phylogeny exerting stronger effects in herbivores and omnivores compared to carnivores (Cheng et al., 2025). In this study, we performed 16S rRNA gene sequencing and functional prediction analyses on 139 horses representing different breeds (Thoroughbreds, hybrids, Mongolian horses, and Warmbloods) and distinct feeding regimens (stabled and grazing). Our findings systematically characterize the structural and functional features of the equine gut microbiota and provide evidence consistent with the view that both host genetic background and feeding management collectively contribute to the observed microbial differences.

### 4.1 Breed dominates the overall structure and diversity of the equine gut microbiota

The four horse groups represented by Mongolian horses, Thoroughbreds, and their hybrids exhibited a highly conserved “nutrient-efficient” gut microbial community structure. These groups demonstrated high alpha diversity, characterized by rich and even species composition. Among them, Mongolian horses harbored the highest richness and diversity of the gut microbiota, a feature potentially attributable to their long-term natural grazing environment and strong local adaptability. The gut microbial diversity of hybrid horses fell between that of Thoroughbreds and Mongolian horses, suggesting that genetic background may influence gut microbial composition in a “dose-dependent” manner. Specifically, the naturally selected indigenous Mongolian horses may have retained a more diverse and enriched gut microbiome adapted to their environment, whereas the highly selectively bred Thoroughbreds may have partially lost such diversity, with their hybrids displaying an intermediate state. Notably, although numerical differences in alpha diversity indices were observed between stabled and grazing hybrid horses, these differences did not reach statistical significance.

The gut microbial diversity of the two Warmblood horse groups was significantly lower than that of the other four groups, a difference that may be attributed to factors such as breed genetic background and management practices. Although the WBH1 (Beijing) and WBH2 (Xing’an League) groups originated from different geographic locations and differed in sample size (5 vs. 11 individuals), the means and degrees of variation of their alpha diversity indices were statistically homogeneous. This observation suggests that, for the Warmblood breed, the influence of breed itself may outweigh the effects of different farm management practices, or alternatively, that the management and feeding practices for Warmblood horses at the two locations were relatively similar. Taken together, these results indicate that breed may play a dominant role in shaping the gut microbiota in the present study. The effects of feeding regimen and geographic location on overall microbial species richness and evenness were smaller than that of breed, suggesting that these factors primarily modulate the specific composition and functional capacities of the microbial community rather than altering overall community diversity.

Extensive studies in mice (Benson et al., 2010), pigs (López-García et al., 2021), cattle (Wang et al., 2024), and sheep (Chang et al., 2020) have consistently shown that breed significantly shapes the gut microbiota. Bao et al. (2022) further reported significant differences in the composition and diversity of gut bacterial communities among Shetland ponies, Mongolian wild asses, and plains zebras. In the present study, analysis based on Bray–Curtis distances revealed distinct structural differences among the fecal bacterial communities of the six horse groups. Notably, the two hybrid groups (HH1 and HH2) exhibited the closest community distance to each other, while also showing relatively close proximity to their parental breeds (TH and MH). Similarly, the two Warmblood groups (WBH1 and WBH2) clustered closely together, and both groups were the most distantly separated from the other four groups. These findings indicate that horses of the same breed—such as HH1 vs. HH2 and WBH1 vs. WBH2—harbor more similar gut microbial structures, whereas significant structural differences exist between distinct breeds.

Our PERMANOVA analysis further quantified the contributions of different factors to gut microbial community variation. Breed emerged as the predominant factor, explaining 44.8% of the total variation (*R*^2^ = 0.448, *p* < 0.001), followed by feeding regimen (10.3%, *p* < 0.001) and geographic location (2.7%, *p* < 0.01). To ensure that the PERMANOVA results were not biased by differences in within-group dispersion, a permutational analysis of multivariate dispersions (PERMDISP) was performed. The PERMDISP test showed no significant differences in multivariate dispersion among the six groups (*F* = 0.559, *p* = 0.732 > 0.05, Supplementary Figure 1), confirming that the observed compositional differences reflect genuine shifts in microbial community centroids rather than heterogeneity in within-group variability. These findings clearly demonstrate that while feeding practices can fine-tune the composition and functional capacities of the gut microbiota, their influence is considerably smaller than that of breed, which is determined by host genetic background. Notably, a direct comparison between stabled (HH1) and grazing (HH2) hybrid horses—which share the same genetic background—revealed that feeding regimen alone explained 13.4% of the variation (*p* < 0.001), providing direct evidence that management practices significantly shape microbial community structure under identical genetic conditions. Furthermore, the lack of a significant difference between Warmblood horses from different geographic locations (Beijing vs. Xing’an League; *R*^2^ = 0.081, *p* = 0.267) further underscores the dominant role of breed in shaping the gut microbiota. This pattern aligns with findings in other livestock species, such as pigs (López-García et al., 2021) and cattle (Wang et al., 2024), where host genetics has been shown to be the primary determinant of core gut microbiota composition.

### 4.2 Phylum-level conservation and breed-specific characteristics

At the phylum level, Thoroughbreds, hybrids, and Mongolian horses shared Firmicutes and Bacteroidota as the absolute dominant phyla (together accounting for >80% of relative abundance), with Verrucomicrobiota, Proteobacteria, Spirochaetota, Actinobacteriota, and Fibrobacterota present as secondary components. This compositional pattern is characteristic of a typical herbivorous gut microbiota, dominated by Firmicutes and Bacteroidota (>80% combined), which is highly consistent with the fiber-rich plant-based diet of horses. These findings are in agreement with previous studies by Zhao et al. (2016) and Wen et al. (2022), who investigated the gut microbial differences between Thoroughbreds and Mongolian horses and reported the same dominant phyla, albeit with different relative abundances and with some variations observed among different groups.

Both Firmicutes and Bacteroidota play essential roles in mammalian digestion and nutrient absorption. The Firmicutes/Bacteroidetes (F/B) ratio is widely recognized as an important indicator for maintaining normal intestinal homeostasis and influencing host nutrient absorption (Stojanov et al., 2020). Consistent with the findings of Wen et al. (2022), who reported that hybrid horses exhibited a higher F/B ratio (3.65) compared to Thoroughbreds (2.20) and Mongolian horses (3.58), the two hybrid groups (2.36 and 2.19) in the present study demonstrated significantly higher F/B ratios than Thoroughbreds (2.10), Mongolian horses (1.19), and the two Warmblood horses groups (1.61 and 1.58, *p* < 0.05). This suggests that hybrid horses may possess a superior capacity for feed nutrient utilization compared to their parental breeds.

Among the four groups, Thoroughbreds harbored the highest relative abundance of Spirochaetota (5.59%), while Mongolian horses exhibited the highest relative abundance of Verrucomicrobiota (13.04%). The gut microbial structure of the hybrid groups (HH1 and HH2) was overall intermediate between their parental breeds (TH and MH), indicating that genetic admixture may lead to functional optimization of the gut microbiota. Notably, the grazing hybrid group (HH2) showed significantly higher relative abundances of Firmicutes (59.01%) and Fibrobacterota (1.6%) compared to both parental breeds (TH and MH) (*p* < 0.05), as well as compared to the stabled hybrid group (HH1) (*p* < 0.05). This transgressive characteristic—exceeding both parental breeds—suggests that grazing hybrids may possess an enhanced capacity for dietary fiber degradation, likely resulting from the combined effects of host genetics and feeding regimen.

### 4.3 Genus-level functional differentiation driven by breed and feeding regimen

At the genus level, the TH, HH1, HH2, and MH groups shared a set of bacterial genera whose core functions are centered on fiber degradation and short-chain fatty acid (SCFA) production. Each group exhibited distinct genus-level distribution patterns characteristic of their breed and feeding regimen.

In the TH group, the absolute dominant genera were *Treponema*, *UCG-002*, *UCG-005*, and *Streptococcus*. *Treponema* is a key fiber-degrading bacterium in the mammalian intestine, participating in the degradation of cellulose and pectin (Pu et al., 2025). *UCG-002* and *UCG-005*, as uncultured members of the Lachnospiraceae and Ruminococcaceae families, are also generally considered to play roles in fiber degradation and butyrate production (Biddle et al., 2013). The combination of these three genera may suggest that the TH group retains a robust capacity for fiber fermentation under stabled conditions. However, a concomitant increase in the relative abundance of *Streptococcus* was also observed in the TH group. *Streptococcus* is a genus known for rapid fermentation of carbohydrates and lactic acid production, and its overgrowth has been associated with high-starch or concentrate-rich diets, potentially leading to decreased intestinal pH and gut dysbiosis (Daly et al., 2012). Therefore, the unique gut microbial structure of the TH group may reflect a mixed metabolic state: on one hand, retaining a strong fiber-degrading capacity, and on the other hand, exhibiting features associated with readily fermentable carbohydrates as a consequence of the stabled feeding regimen.

The stabled hybrid horses (HH1) exhibited a gut microbial structure dominated by the Lachnospiraceae members *Lachnospiraceae_UCG-009*, *Lachnospiraceae_AC2044_group*, and *Akkermansia*. Members of the Lachnospiraceae family are important producers of short-chain fatty acids (SCFAs), particularly butyrate, and play critical roles in maintaining intestinal epithelial health and promoting an anti-inflammatory environment (Vacca et al., 2020). This composition suggests that the HH1 group possesses a strong fiber-degrading capacity and a robust SCFA-producing potential. Akkermansia is a mucin-degrading bacterium that colonizes the mucus layer, and its presence is considered to stimulate mucus regeneration and enhance intestinal barrier function (Everard et al., 2013). This feature may indicate that under stabled conditions, the HH1 gut microbiota, while maintaining efficient nutrient metabolism, may have also developed a more active mucosal metabolic niche. This could represent a relatively stable adaptive characteristic of the stabled environment, distinct from that of natural grazing. Collectively, this highly specialized microbial structure may represent a functional adaptation of the HH1 gut microbiota to the stable, consistent dietary environment under stabled management.

The grazing hybrid horses (HH2) exhibited a unique assemblage of dominant genera, including *Ruminococcus*, *Saccharofermentans*, *unclassified Lachnospiraceae*, *Lachnospiraceae_AC2044_group*, *Fibrobacter, p-251-o5_unclassified*, *Clostridiales_Family_XIV._ Incertae_Sedis_unclassified*, and *Prevotellaceae_UCG-001*. This group harbored the most robust combination of fiber-degrading genera among all groups. *Ruminococcus* and *Fibrobacter* are the most efficient cellulose-degrading genera within the phyla Firmicutes and Fibrobacterota, respectively (Froidurot and Julliand, 2022). Their synergistic action likely constitutes the core force enabling HH2 to efficiently disrupt the physical barrier of plant cell walls. Concurrently, multiple members of the Lachnospiraceae family, along with *Clostridiales_Family_XIV*._ Incertae_Sedis_unclassified (belonging to the order Clostridiales), are key contributors to butyrate or propionate production, likely converting primary degradation products further into butyrate, which is crucial for intestinal health (Louis and Flint, 2017). The Bacteroidota-affiliated genera *p-251-o5_unclassified* and *Prevotellaceae_UCG-001* may specialize in the degradation of hemicellulose and pectin, thereby enriching the diversity of polysaccharide utilization (Wirth et al., 2018). Furthermore, Saccharofermentans, a saccharolytic fermenter, likely utilizes soluble oligosaccharides generated from the degradation system, primarily producing acetate and ensuring efficient carbon flow conversion (El Houari et al., 2023). The microbial community of the HH2 group is not characterized by the dominance of a single genus, but rather represents a functionally complementary, hierarchically organized fiber-degrading consortium. *Ruminococcus* and *Fibrobacter* spearhead the degradation of the most recalcitrant cellulose, *Prevotellaceae_UCG-001* processes other structural polysaccharides, while *Saccharofermentans* and *Lachnospiraceae* members are responsible for downstream sugar fermentation and the conversion of end products, particularly butyrate. This sophisticated “assembly line”-like functional configuration provides a microbial ecological explanation for the transgressive advantage observed in grazing hybrid horses. This configuration not only inherits the fiber-degrading potential of its parental breeds (Mongolian horses and Thoroughbreds) but also specifically enriches and optimizes the interaction network among functional microbial groups under grazing conditions, thereby maximizing the efficiency of energy extraction from roughage.

Under identical genetic backgrounds (both groups being hybrids), feeding regimen drove the functional niche differentiation of the gut microbiota. The grazing group (HH2) exhibited a microbiota more inclined toward the degradation of complex plant fibers, as specifically reflected by the enrichment of genera such as *Ruminococcus* and *Fibrobacter*. In contrast, the stabled group (HH1) demonstrated a microbiota more focused on the efficient processing of readily fermentable carbohydrates and the enhancement of intestinal epithelial nutrient supply, characterized by the enrichment of Lachnospiraceae and related genera. Although this specialization confers metabolic efficiency advantages under stabled conditions, it may also imply reduced microbiota resilience, potentially resulting in a weaker capacity to adapt to sudden dietary changes compared to the more diverse microbiota of grazing horses. These findings provide an important scientific basis for precision nutritional management in stabled horses, emphasizing the need to maintain microbiota stability while providing energy-dense diets.

Mongolian horses (MH, grazing) harbored a distinct set of dominant genera, including *Rikenellaceae_RC9_gut_group*, *NK4A214_group*, *WCHB1-41_unclassified*, *UCG-010_unclassified*, *F082_unclassified*, *Ruminococcaceae_unclassified, [Eubacterium]_coprostanoligenes_group_unclassified*, and *Bacteroidales_RF16_group_unclassified*. The abundance of *Rikenellaceae_RC9_gut_group* is significantly positively correlated with dietary fiber level, positioning it as an important microbial marker for efficient roughage utilization in the host (Wang et al., 2025). By fermenting plant polysaccharides, it primarily produces acetate and propionate, thereby providing energy to the host. Additionally, unclassified Bacteroidales lineages (e.g., *F082_unclassified*, *Bacteroidales_RF16_group_unclassified*) and members of the Ruminococcaceae family (*Ruminococcaceae_unclassified*) collectively constitute a multi-layered, finely divided plant polysaccharide degradation network, ensuring comprehensive utilization of diverse complex carbohydrates from natural vegetation (Louis and Flint, 2017). *NK4A214_group*, a core member of the Oscillospiraceae family within Firmicutes, also possesses robust polysaccharide-degrading capabilities. Along with Clostridiales-affiliated bacteria such as *UCG-010_unclassified*, it is widely recognized as a potential important producer of intestinal butyrate (Konikoff and Gophna, 2016). Butyrate serves as the preferred energy source for colonic epithelial cells and plays an irreplaceable role in maintaining intestinal barrier integrity and an anti-inflammatory environment. In both human and animal studies, its abundance is closely associated with a healthy metabolic state. *[Eubacterium]_coprostanoligenes_group_unclassified* is known for its ability to convert cholesterol into steroids, conferring upon the MH gut microbiota an additional function of modulating host lipid metabolism (Chen et al., 2022). Furthermore, *WCHB1-41_unclassified* has been shown to be significantly enriched in the gut of yaks during cold seasons. Functional analyses have revealed that the arginine and fatty acid anabolic pathways encoded by *Akkermansia*, Kiritimatiellaeota, and WCHB1-41 effectively enhance energy and nitrogen utilization efficiency in yaks, aiding their survival under severe cold conditions (Guo et al., 2021). In summary, the well-balanced gut microbiota of Mongolian horses represents a super microbial consortium that has been optimized through long-term natural selection, characterized by high diversity, strong complementarity, and multifunctionality. It not only maximizes efficiency in fiber degradation and energy conversion but also participates in fine-tuning host physiology through pathways such as steroid metabolism. This complex and robust ecological structure constitutes the microbiological solution enabling Mongolian horses to adapt to the natural grazing environment of the cold plateau.

### 4.4 Distinct gut microbial composition of warmblood horses

The gut microbial structure of Warmblood horses (WBH1 and WBH2) exhibited distinct characteristics. Lan et al. (2025) compared the gut microbiota of Guizhou horses and Dutch Warmblood horses and reported that, at the phylum level, Firmicutes (45.35 and 48.33%, respectively), Bacteroidota (20.82 and 29.93%), and Verrucomicrobiota (12.60 and 8.47%) were the predominant phyla in the two groups. In contrast to the gut microbial features of Dutch Warmblood horses revealed by Lan et al. (2025), the Warmblood horses in the present study displayed a markedly different composition, characterized by an elevated abundance of Proteobacteria (58.76–67.75%), a reduced abundance of Firmicutes (17.55–19.50%) and Bacteroidota (11.13–12.11%), a relatively higher abundance of Actinobacteriota (1.13–6.81%), and a drastically reduced abundance of Verrucomicrobiota (0.95–1.98%).

The Firmicutes/Bacteroidetes (F/B) ratio is often used as an indicator of gut microbial balance. An elevated F/B ratio is typically observed in obese individuals, whereas a decreased ratio has been associated with inflammatory bowel disease (Stojanov et al., 2020). The F/B ratios of Thoroughbreds, hybrid, and Mongolian horses (2.10, 2.36, 2.19, and 1.91) were significantly higher than those of Warmblood horses (1.61 and 1.57, *p* < 0.01). The relatively low F/B ratio and the reduced abundance of fiber-degrading phyla (Firmicutes and Bacteroidota) in Warmblood horses suggest a distinct compositional pattern compared to the other groups. In addition, the low alpha diversity observed in these horses is consistent with this altered community structure.

The gut microbiota of WBH1 (Warmblood horses from Beijing) exhibited a distinctive structure, dominated by *Acinetobacter* (38.74%), *Comamonas* (5.64%), *Solibacillus* (2.78%), *Streptococcus* (2.97%), and *Stenotrophomonas* (2.73%). In stark contrast, the core fiber-degrading genera that should predominate in the gut of healthy herbivores—such as *Rikenellaceae_RC9_gut_group* (0.13%), *Ruminococcus* (0.07%), and *Treponema* (0.06%)—were drastically reduced. WBH2 (Warmblood horses from the Xing’an League) exhibited a similar but more pronounced microbial structure, with the dominant genera being *Stenotrophomonas* (17.60%), *Brevundimonas* (16.25%), *Acinetobacter* (11.80%), and *Comamonas* (9.11%). Notably, compared to WBH1, the WBH2 group showed slight increases in certain potentially beneficial functional genera, such as *Lachnospiraceae_UCG-009* (0.94%) and *Christensenellaceae_R-7_group* (1.36%).

Given that the genera *Acinetobacter*, *Stenotrophomonas, Brevundimonas*, and *Comamonas* are commonly reported as environmental microorganisms (Shin et al., 2015) and, in some circumstances, potential laboratory contaminants, we took several measures to exclude the possibility of contamination. First, negative controls (using sterile water as template) were included in each batch of DNA extraction and PCR amplification, and no detectable 16S rRNA amplification products were observed in any negative control. Second, both Warmblood horse facilities—one in Beijing and the other in Xing’an League—are geographically distant and maintain stringent management practices, particularly the Beijing facility, which imposes rigorous standards for horse health and environmental cleanliness, making contamination during sample collection highly unlikely. Third, to demonstrate that the high abundance of Proteobacteria was not driven by a single outlier sample, we performed an individual-level validation. As shown in Supplementary Figure 1, bar charts illustrating the distribution of Proteobacteria and the aforementioned genera across individual Warmblood samples confirmed that the elevated abundance was consistently observed across multiple individuals within each group, rather than being attributable to a single outlier or sampling artifact. This consistent pattern observed across two geographically distinct facilities further supports the biological authenticity of this finding.

The high abundance of Proteobacteria observed in Warmblood horses may be associated with the genetic background or physiological characteristics of this breed. Proteobacteria constitute a major phylum of Gram-negative bacteria that includes many opportunistic pathogens, and alterations in their abundance have been linked to intestinal inflammation and metabolic disorders in various host species (Rizzatti et al., 2017). However, the functional implications of this compositional shift in Warmblood horses remain to be elucidated, and future studies integrating metabolomic or metagenomic approaches are warranted to clarify the physiological significance of this observation.

All Warmblood horses appeared clinically healthy at the time of sampling. Nevertheless, the observed microbial composition warrants attention. The predominance of Acinetobacter (up to 38.74%) (Carvalheira et al., 2021) and Stenotrophomonas (up to 17.60% in WBH2) (Brooke, 2012) has been reported as a potential indicator of altered gut microbial balance in some studies. The presence of environmental bacteria such as *Comamonas* and *Solibacillus* (Hem et al., 2022; Catozzi et al., 2017) may suggest exposure to environmental factors, though the specific causes remain unclear. The presence of *Streptococcus*, a genus that includes opportunistic pathogens, may further reflect shifts in the intestinal microenvironment. Collectively, the gut microbiota of Warmblood horses exhibited a distinct composition characterized by reduced diversity, a low F/B ratio, and the dominance of environmental-associated genera. These features distinguish them from the other groups examined in this study and warrant further investigation.

Functional prediction analyses revealed differences in predicted metabolic pathways between Warmblood horses and other groups. In both WBH1 and WBH2, the predicted relative abundance of genes associated with Carbohydrate Metabolism was lower than in other groups, consistent with the observed reduction in fiber-degrading bacteria. The predicted abundances of Replication and Repair, Translation, and Nucleotide Metabolism pathways were also decreased, while Xenobiotics Biodegradation and Metabolism showed a relative increase. The predicted abundances of Lipid Metabolism and Signal Transduction pathways were relatively higher in Warmblood horses compared to other groups. However, these findings are based on PICRUSt2 predictions, which reflect functional potential rather than actual functional activity. Therefore, these results should be interpreted as exploratory and hypothesis-generating. Metagenomic, metatranscriptomic, or metabolomic approaches would be required to confirm these functional differences.

In summary, the gut microbiota of Warmblood horses exhibited a distinct composition compared to the other groups, characterized by high Proteobacteria abundance, a low F/B ratio, reduced alpha diversity, and the dominance of environmental-associated genera. While all horses appeared clinically healthy, this unique microbial profile may reflect breed-specific characteristics or the influence of management practices. However, the physiological and health implications of these compositional differences remain unclear and require further investigation in future studies. These findings highlight the need for integrated approaches combining microbiome analysis with physiological and metabolic measurements to better understand the biological significance of these observations.

### 4.5 Combined effects of breed, feeding regimen, and geographic location

Although the six horse groups exhibited significant differences in gut microbial species composition—particularly in the case of Warmblood horses—their macro-level predicted metabolic functional profiles remained relatively similar. This observation is consistent with the concept that microbial communities often maintain stable functional outputs despite taxonomic shifts. However, some functional differences were observed between groups at the predicted pathway level. Warmblood horses showed a trend toward lower predicted abundance of genes associated with basal metabolism and higher predicted abundance of genes associated with xenobiotic metabolism compared to the other groups. These observations are based on PICRUSt2 predictions, which reflect functional potential rather than actual functional activity, and their biological significance requires further validation through independent methods, such as metatranscriptomics or metabolomics. Genus-level analysis revealed fine-scale differences in the taxonomic composition of the gut microbiota. Breed appeared to establish the overall framework of the microbiota, whereas feeding regimen further influenced the composition of specific genera within this framework. The gut microbiota of Warmblood horses was characterized by the loss of core fiber-degrading genera and the dominance of environmental-associated genera, which distinguished them from the other groups in this study. Furthermore, this study systematically characterizes the gut microbial features of the six horse groups and highlights the combined importance of breed, feeding regimen, and geographic location in shaping equine gut microbial diversity and community assembly. Our findings support the substantial role of breed background in structuring microbial communities, while also demonstrating the additional contributions of feeding regimen and geographic location.

### 4.6 Study limitations

Several limitations of this study should be considered when interpreting the results.

First, due to practical constraints in sampling, factors such as breed, geographical location, and feeding regimen were not completely independent in their actual distribution. For instance, horses of different breeds originated from different geographic regions and were managed under feeding regimens adapted to their local conditions. Therefore, caution is warranted when interpreting the relative contributions of these factors. Although PERMANOVA analysis indicated that breed explained a substantial proportion of the variation, this effect may partly incorporate the influence of geographical location and feeding regimen.

Second, the sample size for Warmblood horses was relatively modest, and these animals came from two different geographic sources. This may to some extent affect the ability to characterize within-breed variation, and the related findings should be further validated in studies with larger sample sizes.

Third, this study was primarily based on 16S rRNA gene sequencing, with functional predictions performed using PICRUSt2. This approach reflects the functional potential of the microbial community rather than actual functional activity. Accordingly, interpretations related to function should be considered exploratory, and future studies incorporating metatranscriptomic or metabolomic approaches are needed for validation.

Fourth, all horses in this study were male and concentrated within a narrow age range (2–8 years). While this helps control for potential confounding effects of sex and age, it also means that generalization of the findings to mares or horses of other age groups should be made with caution.

In summary, despite these limitations, this study provides valuable preliminary data for understanding the effects of breed and feeding regimen on the equine gut microbiome and lays the groundwork for more systematic investigations in the future.

## 5 Conclusions and future perspectives

By integrating microbial ecology and bioinformatics analyses, this study systematically elucidated substantial differences in the structure, composition, and predicted functional potential of the gut microbiota among different horse breeds. Our findings suggest that breed is a major factor shaping the structural and compositional landscape of the equine gut microbiota, with its effects being more pronounced than those of feeding regimen and geographic origin within the scope of this study. Notably, we characterized a distinct ecological profile in the gut microbiota of Warmblood horses. This profile is characterized by an elevated abundance of opportunistic associated pathogens, reduced abundance of core nutrient-metabolic related functions, and relatively higher predicted xenobiotic-degrading capacity, suggesting that these horses exhibit a unique microbial composition that differs markedly from the other groups. In contrast, native grazing horses, represented by Mongolian horses, harbor a more balanced microbial community structure. These findings provide new insights into the interplay between host genetic background and the gut microbial ecosystem and offer crucial theoretical foundations and practical guidance for breed-specific health management, precision nutritional intervention, and science-based breeding of horses. Future studies should employ multi-omics technologies and experimental approaches to further validate the underlying mechanisms and to explore strategies for translating these microbiological discoveries into effective practices that enhance equine welfare and performance.

## Data Availability

The raw 16S rRNA gene sequencing data supporting the findings of this study have been deposited in the NCBI under BioProject accession number PRJNA1490074 and are publicly available at https://www.ncbi.nlm.nih.gov/bioproject/PRJNA1490074.
