# Supplementary material for: Differential shaping of equine gut microbiota structure and function by breed and feeding regimen
Source: Front Microbiol. 2026 Jul 16;17:1852554. doi: 10.3389/fmicb.2026.1852554 (PMC13430720; doi:10.3389/fmicb.2026.1852554)
Supplement: Supplementary file 1 [file Supplementary_file_1.docx]

Supplementary Material

# Supplementary Data

A total of 2 supplementary tables and 2 supplementary figures have been added.

# Supplementary Tables

**Supplementary Table S1. Detailed metadata of all sampled horses**

| **Sample ID** | **Group** | **Breed** | **Location** | **Feeding Regimen** | **Sex** | **Age (years)** | **BCS** | **Sampling Date** |
| --- | --- | --- | --- | --- | --- | --- | --- | --- |
| Th1-Th30 | TH | Thoroughbred | Hohhot | Stabled | Male | 2-5 | 5-6 | 2020.6.27 |
| HH1_1-HH1_31 | HH1 | Hybrid (TB×MG) | Hohhot | Stabled | Male | 2-4 | 5-6 | 2020.8.29 |
| HH2_1-HH2_30 | HH2 | Hybrid (TB×MG) | Xilingol League | Grazing | Male | 2-3 | 5-7 | 2020.6.27 |
| MH1-MH32 | MH | Mongolian | Xilingol League | Grazing | Male | 3-5 | 5-7 | 2020.6.27 |
| WBH1-1-WBH1-5 | WBH1 | Warmblood | Beijing | Stabled | Male | 3-8 | 5-6 | 2020.6.12 |
| WBH2-1-WBH2-11 | WBH2 | Warmblood | Xing'an League | Stabled | Male | 2-5 | 5-7 | 2020.8.25 |

**Note:**BCS = Body Condition Score (Henneke 1-9 scale); TB = Thoroughbred; MG = Mongolian.

**Supplementary Table S2. Sequencing data quality control and processing metrics for each sample**

| **Sample** | **Raw_Tags** | **Raw_Bases** | **Valid_Tags** | **Valid_Bases** | **Valid%** | **Q20%** | **Q30%** | **GC%** | **feature_number** |
| --- | --- | --- | --- | --- | --- | --- | --- | --- | --- |
| Th1 | 57643 | 23.10M | 57643 | 23.10M | 100.00 | 95.66 | 88.27 | 52.40 | 1034 |
| Th2 | 74066 | 30.62M | 74066 | 30.62M | 100.00 | 97.08 | 91.60 | 52.46 | 1777 |
| Th3 | 73316 | 30.17M | 73316 | 30.17M | 100.00 | 96.82 | 91.09 | 52.62 | 1884 |
| Th4 | 76104 | 31.50M | 76104 | 31.50M | 100.00 | 96.59 | 90.61 | 52.49 | 1734 |
| Th5 | 71305 | 29.48M | 71305 | 29.48M | 100.00 | 96.52 | 90.16 | 52.64 | 1584 |
| Th6 | 75323 | 31.10M | 75323 | 31.10M | 100.00 | 97.16 | 91.95 | 52.53 | 1937 |
| Th7 | 74337 | 30.77M | 74337 | 30.77M | 100.00 | 97.11 | 91.77 | 52.51 | 1889 |
| Th8 | 60665 | 25.07M | 60665 | 25.07M | 100.00 | 91.00 | 79.04 | 53.03 | 275 |
| Th9 | 76164 | 31.48M | 76164 | 31.48M | 100.00 | 95.55 | 88.06 | 52.92 | 1479 |
| Th10 | 70794 | 29.13M | 70794 | 29.13M | 100.00 | 97.01 | 91.42 | 52.38 | 1459 |
| Th11 | 74199 | 30.62M | 74199 | 30.62M | 100.00 | 96.89 | 91.18 | 52.56 | 1756 |
| Th12 | 71517 | 29.59M | 71517 | 29.59M | 100.00 | 95.89 | 88.78 | 52.46 | 1344 |
| Th13 | 74464 | 30.76M | 74464 | 30.76M | 100.00 | 96.04 | 89.00 | 52.62 | 1310 |
| Th14 | 62278 | 26.04M | 62278 | 26.04M | 100.00 | 96.67 | 90.87 | 52.23 | 1275 |
| Th15 | 70951 | 29.29M | 70951 | 29.29M | 100.00 | 97.13 | 91.72 | 52.22 | 1558 |
| Th16 | 67627 | 27.98M | 67627 | 27.98M | 100.00 | 93.15 | 82.89 | 52.71 | 604 |
| Th17 | 71747 | 29.74M | 71747 | 29.74M | 100.00 | 95.64 | 88.22 | 52.90 | 1409 |
| Th18 | 70590 | 29.06M | 70590 | 29.06M | 100.00 | 97.03 | 91.50 | 53.40 | 1553 |
| Th19 | 76326 | 31.49M | 76326 | 31.49M | 100.00 | 96.64 | 90.66 | 52.68 | 1449 |
| Th20 | 66536 | 27.25M | 66536 | 27.25M | 100.00 | 95.73 | 88.49 | 53.04 | 1070 |
| Th21 | 71522 | 29.56M | 71522 | 29.56M | 100.00 | 96.35 | 89.70 | 52.69 | 1151 |
| Th22 | 69816 | 28.81M | 69816 | 28.81M | 100.00 | 96.87 | 91.23 | 52.78 | 1514 |
| Th23 | 72874 | 30.06M | 72874 | 30.06M | 100.00 | 97.06 | 91.63 | 52.68 | 1440 |
| Th24 | 64237 | 26.35M | 64237 | 26.35M | 100.00 | 92.50 | 81.66 | 53.04 | 403 |
| Th25 | 76640 | 31.56M | 76640 | 31.56M | 100.00 | 96.43 | 90.06 | 53.15 | 1502 |
| Th26 | 72933 | 30.08M | 72933 | 30.08M | 100.00 | 96.98 | 91.31 | 52.93 | 1552 |
| Th27 | 74901 | 30.84M | 74901 | 30.84M | 100.00 | 96.87 | 91.13 | 52.74 | 1800 |
| Th28 | 78595 | 32.43M | 78595 | 32.43M | 100.00 | 96.72 | 90.82 | 52.58 | 1690 |
| Th29 | 79315 | 32.66M | 79315 | 32.66M | 100.00 | 96.77 | 90.71 | 52.72 | 1649 |
| Th30 | 75817 | 31.30M | 75817 | 31.30M | 100.00 | 96.77 | 90.91 | 52.67 | 1839 |
| HH1_1 | 72528 | 29.75M | 72528 | 29.75M | 100.00 | 96.91 | 91.23 | 52.73 | 1586 |
| HH1_2 | 66553 | 27.44M | 66553 | 27.44M | 100.00 | 93.93 | 84.74 | 52.63 | 730 |
| HH1_3 | 72939 | 30.03M | 72939 | 30.03M | 100.00 | 95.26 | 87.45 | 52.46 | 1064 |
| HH1_4 | 75134 | 30.91M | 75134 | 30.91M | 100.00 | 96.90 | 91.18 | 52.36 | 1724 |
| HH1_5 | 72670 | 29.78M | 72670 | 29.78M | 100.00 | 96.37 | 90.02 | 52.34 | 1219 |
| HH1_6 | 65011 | 26.93M | 65011 | 26.93M | 100.00 | 94.95 | 87.06 | 52.78 | 1050 |
| HH1_7 | 76076 | 31.32M | 76076 | 31.32M | 100.00 | 96.15 | 89.28 | 52.69 | 1565 |
| HH1_8 | 67971 | 28.01M | 67971 | 28.01M | 100.00 | 96.00 | 89.07 | 52.91 | 1410 |
| HH1_9 | 75686 | 31.30M | 75686 | 31.30M | 100.00 | 96.66 | 90.64 | 53.15 | 1890 |
| HH1_10 | 65709 | 27.03M | 65709 | 27.03M | 100.00 | 91.79 | 80.40 | 53.67 | 306 |
| HH1_11 | 68934 | 28.29M | 68934 | 28.29M | 100.00 | 95.17 | 87.21 | 52.90 | 1192 |
| HH1_12 | 70247 | 28.80M | 70247 | 28.80M | 100.00 | 96.93 | 91.29 | 52.73 | 1675 |
| HH1_13 | 68667 | 28.27M | 68667 | 28.27M | 100.00 | 94.44 | 85.66 | 53.00 | 913 |
| HH1_14 | 76215 | 31.39M | 76215 | 31.39M | 100.00 | 96.66 | 90.72 | 52.68 | 1922 |
| HH1_15 | 72350 | 29.90M | 72350 | 29.90M | 100.00 | 94.31 | 85.30 | 53.48 | 968 |
| HH1_16 | 67967 | 27.96M | 67967 | 27.96M | 100.00 | 96.55 | 90.51 | 52.70 | 1569 |
| HH1_17 | 72508 | 29.77M | 72508 | 29.77M | 100.00 | 97.08 | 91.68 | 52.89 | 1921 |
| HH1_18 | 63385 | 26.18M | 63385 | 26.18M | 100.00 | 92.38 | 81.45 | 53.14 | 402 |
| HH1_19 | 76698 | 31.52M | 76698 | 31.52M | 100.00 | 97.63 | 93.04 | 52.42 | 2154 |
| HH1_20 | 73963 | 30.61M | 73963 | 30.61M | 100.00 | 96.83 | 91.16 | 53.20 | 2002 |
| HH1_21 | 79821 | 33.14M | 79821 | 33.14M | 100.00 | 96.67 | 90.49 | 53.56 | 1793 |
| HH1_22 | 70172 | 28.75M | 70172 | 28.75M | 100.00 | 96.55 | 90.49 | 52.71 | 1485 |
| HH1_23 | 82630 | 34.10M | 82630 | 34.10M | 100.00 | 96.32 | 89.77 | 53.22 | 1998 |
| HH1_24 | 74004 | 30.54M | 74004 | 30.54M | 100.00 | 96.67 | 90.77 | 53.52 | 1770 |
| HH1_25 | 71626 | 29.57M | 71626 | 29.57M | 100.00 | 96.69 | 90.72 | 52.41 | 1524 |
| HH1_26 | 61382 | 25.33M | 61382 | 25.33M | 100.00 | 92.00 | 80.80 | 53.09 | 350 |
| HH1_27 | 75597 | 31.09M | 75597 | 31.09M | 100.00 | 96.15 | 89.44 | 53.22 | 1804 |
| HH1_28 | 72634 | 30.11M | 72634 | 30.11M | 100.00 | 96.11 | 89.14 | 52.34 | 1477 |
| HH1_29 | 70470 | 29.14M | 70470 | 29.14M | 100.00 | 94.38 | 85.54 | 52.80 | 990 |
| HH1_30 | 73817 | 30.49M | 73817 | 30.49M | 100.00 | 95.62 | 88.09 | 52.93 | 1302 |
| HH1_31 | 69660 | 28.52M | 69660 | 28.52M | 100.00 | 96.07 | 89.10 | 52.70 | 1234 |
| MH1 | 74907 | 30.89M | 74907 | 30.89M | 100.00 | 96.74 | 90.91 | 53.13 | 1900 |
| MH2 | 73455 | 30.34M | 73455 | 30.34M | 100.00 | 96.92 | 91.29 | 53.52 | 1968 |
| MH3 | 65754 | 27.12M | 65754 | 27.12M | 100.00 | 92.30 | 81.26 | 53.79 | 357 |
| MH4 | 70603 | 28.97M | 70603 | 28.97M | 100.00 | 95.31 | 87.48 | 53.04 | 1227 |
| MH5 | 76270 | 31.52M | 76270 | 31.52M | 100.00 | 96.58 | 90.35 | 52.64 | 1656 |
| MH6 | 72354 | 29.68M | 72354 | 29.68M | 100.00 | 96.77 | 91.02 | 53.09 | 1948 |
| MH7 | 75549 | 31.20M | 75549 | 31.20M | 100.00 | 96.21 | 89.73 | 53.18 | 1610 |
| MH8 | 74949 | 30.79M | 74949 | 30.79M | 100.00 | 96.01 | 88.99 | 53.10 | 1478 |
| MH9 | 70377 | 28.99M | 70377 | 28.99M | 100.00 | 96.24 | 89.56 | 53.02 | 1664 |
| MH10 | 70418 | 29.11M | 70418 | 29.11M | 100.00 | 96.60 | 90.44 | 53.39 | 1782 |
| MH11 | 68619 | 28.27M | 68619 | 28.27M | 100.00 | 93.32 | 83.29 | 53.64 | 634 |
| MH12 | 70062 | 28.78M | 70062 | 28.78M | 100.00 | 95.25 | 87.39 | 52.75 | 1038 |
| MH13 | 73076 | 30.06M | 73076 | 30.06M | 100.00 | 96.86 | 91.09 | 52.95 | 1896 |
| MH14 | 75542 | 31.14M | 75542 | 31.14M | 100.00 | 96.59 | 90.56 | 53.04 | 1787 |
| MH15 | 75821 | 31.27M | 75821 | 31.27M | 100.00 | 96.35 | 90.06 | 52.74 | 1677 |
| MH16 | 74763 | 30.21M | 74763 | 30.21M | 100.00 | 96.95 | 91.36 | 52.19 | 1688 |
| MH17 | 72365 | 29.69M | 72365 | 29.69M | 100.00 | 96.84 | 91.19 | 52.93 | 1930 |
| MH18 | 68191 | 28.12M | 68191 | 28.12M | 100.00 | 93.05 | 82.67 | 53.03 | 535 |
| MH19 | 60857 | 25.14M | 60857 | 25.14M | 100.00 | 92.45 | 81.64 | 53.48 | 388 |
| MH20 | 72333 | 29.77M | 72333 | 29.77M | 100.00 | 94.89 | 86.59 | 52.64 | 1175 |
| MH21 | 75546 | 31.10M | 75546 | 31.10M | 100.00 | 96.78 | 90.72 | 52.94 | 1837 |
| MH22 | 75106 | 30.91M | 75106 | 30.91M | 100.00 | 96.49 | 90.40 | 53.04 | 1688 |
| MH23 | 71507 | 29.53M | 71507 | 29.53M | 100.00 | 95.66 | 88.53 | 52.77 | 1327 |
| MH24 | 70293 | 28.95M | 70293 | 28.95M | 100.00 | 95.67 | 88.20 | 53.09 | 1228 |
| MH25 | 73849 | 30.60M | 73849 | 30.60M | 100.00 | 96.61 | 90.74 | 53.43 | 1663 |
| MH26 | 73223 | 30.20M | 73223 | 30.20M | 100.00 | 96.64 | 90.62 | 52.95 | 1726 |
| MH27 | 58169 | 23.97M | 58169 | 23.97M | 100.00 | 90.46 | 78.30 | 53.63 | 177 |
| MH28 | 69462 | 28.58M | 69462 | 28.58M | 100.00 | 95.14 | 87.11 | 52.66 | 1075 |
| MH29 | 73026 | 29.98M | 73026 | 29.98M | 100.00 | 96.83 | 91.03 | 52.45 | 1619 |
| MH30 | 75873 | 31.13M | 75873 | 31.13M | 100.00 | 96.55 | 90.47 | 53.08 | 1868 |
| MH31 | 70353 | 28.90M | 70353 | 28.90M | 100.00 | 95.77 | 88.72 | 53.05 | 1501 |
| MH32 | 73828 | 30.41M | 73828 | 30.41M | 100.00 | 96.00 | 88.95 | 52.80 | 1532 |
| HH2_1 | 73518 | 30.25M | 73518 | 30.25M | 100.00 | 96.45 | 90.27 | 52.71 | 1500 |
| HH2_2 | 69183 | 28.22M | 69183 | 28.22M | 100.00 | 96.12 | 89.45 | 53.06 | 1288 |
| HH2_3 | 72025 | 29.61M | 72025 | 29.61M | 100.00 | 96.08 | 89.17 | 52.65 | 1409 |
| HH2_4 | 74113 | 30.44M | 74113 | 30.44M | 100.00 | 96.77 | 91.05 | 52.75 | 1764 |
| HH2_5 | 67235 | 27.66M | 67235 | 27.66M | 100.00 | 96.42 | 90.00 | 52.71 | 1430 |
| HH2_6 | 60742 | 24.93M | 60742 | 24.93M | 100.00 | 92.63 | 81.87 | 53.10 | 356 |
| HH2_7 | 69053 | 28.50M | 69053 | 28.50M | 100.00 | 93.55 | 83.82 | 52.90 | 695 |
| HH2_8 | 73941 | 30.57M | 73941 | 30.57M | 100.00 | 96.07 | 89.06 | 52.67 | 1349 |
| HH2_9 | 71489 | 29.29M | 71489 | 29.29M | 100.00 | 96.36 | 89.92 | 52.71 | 1420 |
| HH2_10 | 64771 | 26.56M | 64771 | 26.56M | 100.00 | 92.76 | 82.97 | 52.69 | 470 |
| HH2_11 | 70187 | 28.79M | 70187 | 28.79M | 100.00 | 95.30 | 87.33 | 53.22 | 1067 |
| HH2_12 | 72322 | 30.00M | 72322 | 30.00M | 100.00 | 96.67 | 90.69 | 52.73 | 1770 |
| HH2_13 | 70492 | 29.18M | 70492 | 29.18M | 100.00 | 96.73 | 90.65 | 52.82 | 1774 |
| HH2_14 | 58492 | 24.15M | 58492 | 24.15M | 100.00 | 90.56 | 78.28 | 53.13 | 190 |
| HH2_15 | 70240 | 28.98M | 70240 | 28.98M | 100.00 | 95.50 | 87.87 | 52.75 | 1221 |
| HH2_16 | 75138 | 30.76M | 75138 | 30.76M | 100.00 | 96.93 | 91.22 | 52.78 | 1823 |
| HH2_17 | 72961 | 29.94M | 72961 | 29.94M | 100.00 | 96.81 | 91.04 | 52.51 | 1615 |
| HH2_18 | 71490 | 29.38M | 71490 | 29.38M | 100.00 | 96.57 | 90.50 | 52.98 | 1767 |
| HH2_19 | 73656 | 30.26M | 73656 | 30.26M | 100.00 | 96.34 | 89.68 | 52.51 | 1376 |
| HH2_20 | 70683 | 29.11M | 70683 | 29.11M | 100.00 | 97.01 | 91.53 | 52.67 | 1895 |
| HH2_21 | 74920 | 30.66M | 74920 | 30.66M | 100.00 | 97.11 | 91.77 | 52.82 | 1990 |
| HH2_22 | 62405 | 25.68M | 62405 | 25.68M | 100.00 | 91.93 | 80.59 | 52.86 | 337 |
| HH2_23 | 69275 | 28.41M | 69275 | 28.41M | 100.00 | 94.77 | 86.26 | 52.67 | 862 |
| HH2_24 | 72110 | 29.53M | 72110 | 29.53M | 100.00 | 96.71 | 90.66 | 52.23 | 1423 |
| HH2_25 | 74353 | 30.53M | 74353 | 30.53M | 100.00 | 96.58 | 90.48 | 52.76 | 1652 |
| HH2_26 | 70843 | 29.11M | 70843 | 29.11M | 100.00 | 95.77 | 88.46 | 52.83 | 1383 |
| HH2_27 | 71872 | 29.41M | 71872 | 29.41M | 100.00 | 95.98 | 88.85 | 52.91 | 1208 |
| HH2_28 | 79944 | 32.88M | 79944 | 32.88M | 100.00 | 97.00 | 91.33 | 52.78 | 1854 |
| HH2_29 | 67358 | 27.69M | 67358 | 27.69M | 100.00 | 96.80 | 90.85 | 52.56 | 1562 |
| HH2_30 | 62114 | 25.49M | 62114 | 25.49M | 100.00 | 92.52 | 81.66 | 53.48 | 373 |
| WBH1-1 | 75338 | 31.73M | 75338 | 31.73M | 100.00 | 95.44 | 87.80 | 52.08 | 430 |
| WBH1-2 | 69223 | 29.28M | 69223 | 29.28M | 100.00 | 95.89 | 89.12 | 51.77 | 466 |
| WBH1-3 | 74109 | 31.02M | 74109 | 31.02M | 100.00 | 94.70 | 86.37 | 52.17 | 722 |
| WBH1-4 | 72243 | 30.65M | 72243 | 30.65M | 100.00 | 95.10 | 87.13 | 51.61 | 288 |
| WBH1-5 | 80273 | 33.60M | 80273 | 33.60M | 100.00 | 96.15 | 89.78 | 53.39 | 541 |
| WBH2-1 | 67265 | 27.58M | 67265 | 27.58M | 100.00 | 97.04 | 91.60 | 52.45 | 475 |
| WBH2-2 | 59184 | 25.20M | 59184 | 25.20M | 100.00 | 95.96 | 89.52 | 51.36 | 248 |
| WBH2-3 | 78425 | 33.25M | 78425 | 33.25M | 100.00 | 96.68 | 90.76 | 51.84 | 393 |
| WBH2-4 | 75622 | 31.75M | 75622 | 31.75M | 100.00 | 96.00 | 89.22 | 52.49 | 351 |
| WBH2-5 | 66521 | 28.30M | 66521 | 28.30M | 100.00 | 95.94 | 89.35 | 51.29 | 331 |
| WBH2-6 | 73512 | 31.29M | 73512 | 31.29M | 100.00 | 94.83 | 86.53 | 50.48 | 90 |
| WBH2-7 | 71355 | 29.96M | 71355 | 29.96M | 100.00 | 95.82 | 88.65 | 52.76 | 220 |
| WBH2-8 | 68013 | 28.45M | 68013 | 28.45M | 100.00 | 96.90 | 91.29 | 51.63 | 433 |
| WBH2-9 | 74709 | 31.13M | 74709 | 31.13M | 100.00 | 97.06 | 91.57 | 52.85 | 527 |
| WBH2-10 | 72247 | 29.82M | 72247 | 29.82M | 100.00 | 96.89 | 91.18 | 52.82 | 1185 |
| WBH2-11 | 64512 | 26.84M | 64512 | 26.84M | 100.00 | 96.92 | 91.41 | 52.42 | 309 |

**Note:** Raw_Reads, number of raw reads; Raw_Bases, total raw bases (M); Valid_Tags, number of valid merged reads after preprocessing; Valid_Bases, total valid bases (M); Valid%, percentage of valid data relative to raw data; Q20%, percentage of valid data with quality ≥ Q20; Q30%, percentage of valid data with quality ≥ Q30; GC%, GC content of valid data. feature_number, number of features (ASVs) calculated for each sample or group; features with non-zero abundance across all three groups were defined as shared, while features present in only one sample or group were defined as unique.

## Supplementary Figures

PERMDISP analysis

To assess whether the observed differences in microbial community structure among groups were influenced by differences in within-group dispersion, permutational analysis of multivariate dispersions (PERMDISP) was performed. PERMDISP calculates the distance from each sample to the centroid of its respective group in the multivariate space and compares the average distances among groups. The analysis was based on the Bray-Curtis distance matrix with 999 permutations. A non-significant PERMDISP result (p > 0.05) indicates that the groups have comparable within-group variability, confirming that the PERMANOVA results reflect true differences in community composition rather than differences in dispersion. PERMDISP analysis was implemented using the betadisper function in the R package vegan (v2.6-4).


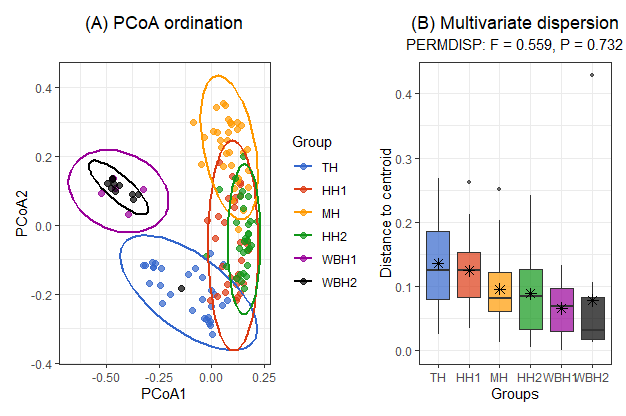


**Supplementary Figure 1.** Permutational analysis of multivariate dispersions (PERMDISP) based on PCoA coordinates. (A) PCoA ordination of individual samples with group centroids, illustrating the spatial distribution of each group. (B) Boxplot showing the distances from each sample to its group centroid, with boxes representing the median and interquartile range.

**
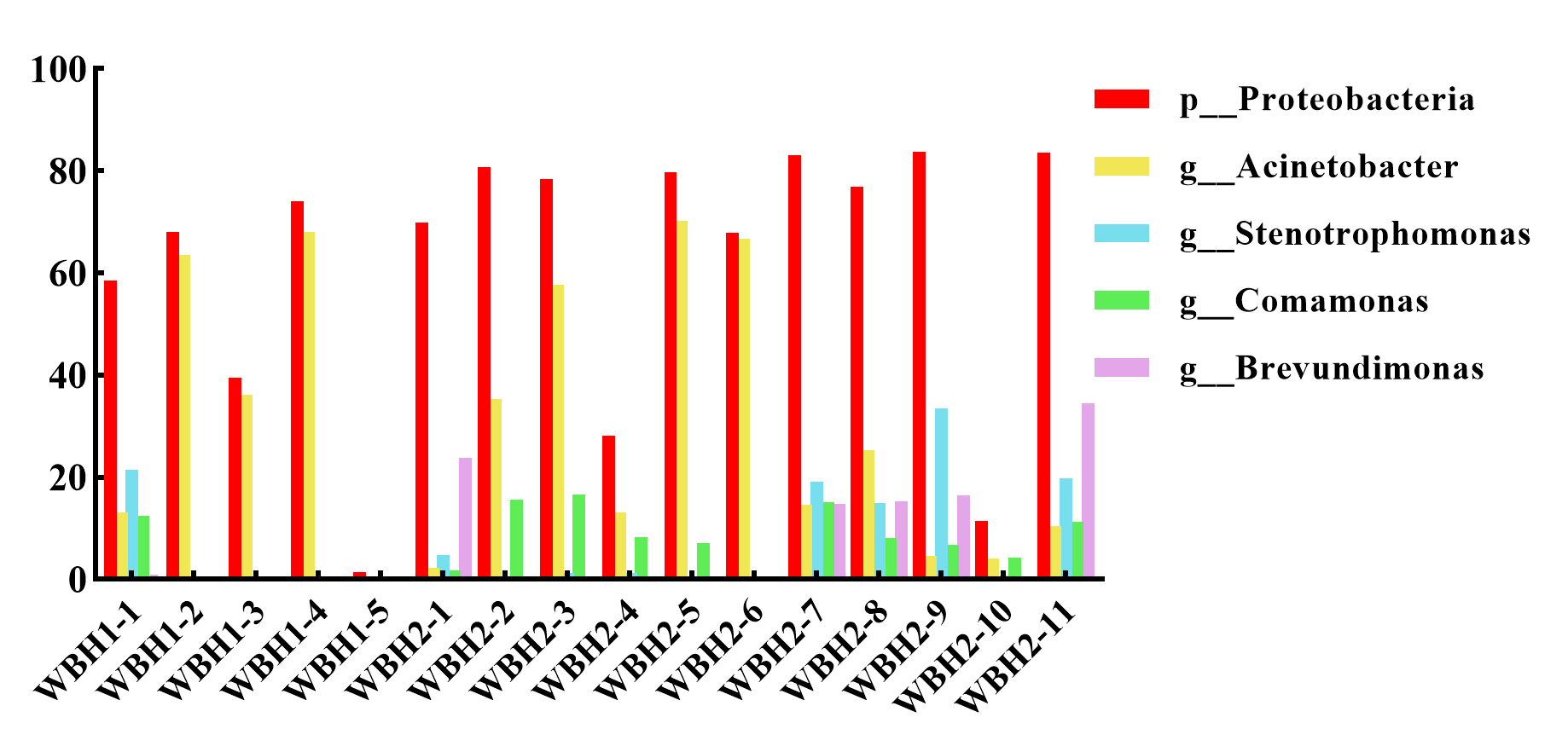
**

**Supplementary Figure 2.** Individual-level abundance distribution of phylum Proteobacteria and representative environmental opportunistic genera (Acinetobacter, Stenotrophomonas, Comamonas, and Brevundimonas) in Warmblood horses (WBH1, n = 5; WBH2, n = 11)
